# Tuberculosis Skin Test for the Diagnosis of Pediatric Tuberculosis: Comparison with Tuberculin Skin Test and Interferon-Gamma Release Assays

**DOI:** 10.3390/microorganisms14050974

**Published:** 2026-04-26

**Authors:** Susanna Esposito, Beatrice Rita Campana, Gaia Giorgia Arnesano, Nicola Principi

**Affiliations:** 1 Pediatric Clinic, Department of Medicine and Surgery, University of Parma, 43126 Parma, Italygaiagiorgia.arnesano@unipr.it (G.G.A.); 2 Università degli Studi di Milano, 20122 Milan, Italy; nicola.principi@unimi.it

**Keywords:** pediatric tuberculosis, TBST, TST, IGRA, *Mycobacterium tuberculosis*, latent tuberculosis infection

## Abstract

Tuberculosis (TB) remains a leading cause of morbidity and mortality worldwide, with children representing a particularly vulnerable population in whom diagnosis is often challenging. Pediatric TB is typically paucibacillary and presents with non-specific clinical manifestations, limiting the sensitivity of microbiological confirmation and increasing reliance on immunological tests. The Tuberculin Skin Test (TST) and Interferon-Gamma Release Assays (IGRAs) are the most widely used tools for detecting *Mycobacterium tuberculosis* infection, yet both have important limitations, especially in young children and in Bacillus Calmette–Guérin (BCG)-vaccinated populations. TST lacks specificity due to cross-reactivity with BCG and environmental mycobacteria, while IGRAs, although more specific, require laboratory infrastructure and may have reduced sensitivity in early childhood. The Tuberculosis Skin Test (TBST), based on *M. tuberculosis*-specific antigens such as ESAT-6 and CFP-10, has emerged as a promising alternative that combines the operational simplicity of TST with the antigenic specificity of IGRA. This paper reviews the immunological principles, diagnostic performance, and practical considerations of TBST in pediatric populations, with direct comparison to TST and IGRA. Evidence from recent studies suggests that TBST may offer improved specificity over TST, with broadly comparable diagnostic accuracy to IGRA in some settings, although findings are not fully consistent across studies. Particular attention is given to its performance in BCG-vaccinated children and, based on emerging evidence, in those under five years of age. The potential role of TBST in clinical algorithms and public health strategies is discussed, along with current evidence gaps and future research priorities.

## 1. Introduction

Tuberculosis (TB) continues to represent one of the most significant infectious disease threats globally, despite decades of control efforts [1,2]. According to the World Health Organization (WHO), an estimated 10 million individuals develop TB each year, with children accounting for more than one million of these cases [1]. Pediatric TB contributes substantially to global morbidity and mortality, particularly in low- and middle-income countries where access to diagnostic and healthcare services remains limited [3]. Importantly, TB in children is often underdiagnosed and underreported, leading to a significant gap between estimated incidence and notified cases [1].

The diagnosis of tuberculosis in children presents unique and well-recognized challenges [4]. Unlike adults, children frequently develop paucibacillary disease, which limits the sensitivity of microbiological methods such as smear microscopy, culture, and nucleic acid amplification tests [4]. Furthermore, young children are often unable to produce sputum samples, and alternative specimen collection methods (e.g., gastric aspirates or induced sputum) are invasive and not always feasible in routine clinical practice [5]. As a result, the diagnosis of pediatric TB often relies on a combination of clinical, radiological, epidemiological, and immunological evidence rather than bacteriological confirmation [4].

Immunological tests that detect host responses to *Mycobacterium tuberculosis* (Mtb) antigens play a central role in the diagnosis of latent TB infection (LTBI) and serve as supportive tools in the diagnosis of active disease [6,7]. The Tuberculin Skin Test (TST), introduced over a century ago, remains widely used due to its low cost and operational simplicity [8]. However, TST suffers from important limitations, including low specificity in Bacillus Calmette–Guérin (BCG)-vaccinated individuals and cross-reactivity with non-tuberculous mycobacteria [8,9]. In addition, its sensitivity may be reduced in young children, malnourished individuals, and those with immunosuppression [7].

Interferon-Gamma Release Assays (IGRAs) were developed to overcome some of these limitations by using Mtb-specific antigens such as ESAT-6 and CFP-10 [6,10]. These tests offer improved specificity compared to TST and are not affected by prior BCG vaccination [6]. Nevertheless, IGRAs require blood sampling, laboratory infrastructure, and timely sample processing, which limit their accessibility in many high-burden settings [11]. Moreover, their performance in very young children remains suboptimal, with higher rates of indeterminate results and variable sensitivity [12].

In response to these challenges, the Tuberculosis Skin Test (TBST), also known as recombinant antigen-based skin tests (e.g., C-Tb, Diaskintest), has emerged as a novel diagnostic approach [13,14,15]. TBST utilizes the same Mtb-specific antigens as IGRAs but is administered intradermally, eliciting a delayed-type hypersensitivity response similar to TST [13]. This innovative strategy aims to combine the high specificity of IGRA with the simplicity, low cost, and field applicability of TST.

Early studies have demonstrated promising results for TBST, particularly in BCG-vaccinated populations where traditional TST performance is compromised [13,14,15,16,17]. In addition, TBST may offer advantages in pediatric populations due to its in vivo mechanism, which could be more robust in young children compared to ex vivo assays [14]. However, evidence remains heterogeneous, and questions persist regarding its sensitivity in different age groups, its performance in immunocompromised children (including those with HIV), and its role within existing diagnostic algorithms [7].

Given the urgent need for improved diagnostic tools in pediatric TB, a comprehensive evaluation of TBST is both timely and necessary. This paper aims to critically assess the role of TBST in the diagnosis of pediatric TB by comparing its immunological basis, diagnostic accuracy, operational characteristics, and clinical applicability with those of TST and IGRA. By synthesizing current evidence, this review seeks to clarify whether TBST can address existing gaps in pediatric TB diagnostics and contribute meaningfully to global TB control strategies.

## 2. Methods

This work is a narrative review with elements of a structured literature search aimed at synthesizing current evidence on the use of TBST in the diagnosis of pediatric TB and comparing its performance with TST and IGRAs. The review focuses on studies involving children and adolescents (0–18 years), including both LTBI and active TB disease.

A literature search was conducted across major electronic databases, including PubMed/MEDLINE, Embase, and Scopus, covering publications from January 2005 to December 2025. Search terms included combinations of keywords and Medical Subject Headings (MeSH), such as: “tuberculosis”, “pediatric”, “children”, “tuberculin skin test”, “TST”, “interferon gamma release assay”, “IGRA”, “ESAT-6”, “CFP-10”, “C-Tb”, “Diaskintest”, and “TB skin test”. Boolean operators (AND, OR) were used to refine the search.

Reference lists of relevant articles and reviews were also screened to identify additional eligible studies. Only articles published in English were included.

Studies were included if they met the following criteria: involved pediatric populations (0–18 years); evaluated TBST, TST, IGRA, or direct comparisons among these tests; reported diagnostic performance outcomes (e.g., sensitivity, specificity, predictive values); and included either confirmed TB cases, suspected TB, or screening populations. Exclusion criteria were as follows: studies conducted exclusively in adult populations; case reports or small case series (*n* < 10); and non-original articles (e.g., editorials, commentaries) unless used for contextual discussion.

Data were extracted independently from selected studies and included study design, population characteristics, type of diagnostic test, reference standard, and reported outcomes (sensitivity, specificity, concordance rates). Where available, subgroup analyses in young children (<5 years), BCG-vaccinated populations, and immunocompromised patients were also recorded.

Given the heterogeneity of study designs and reference standards, a quantitative meta-analysis was not performed. Instead, findings were synthesized narratively, with emphasis on consistency across studies and clinically relevant differences between diagnostic methods.

For the purposes of this review, active TB was defined according to microbiological confirmation or clinical diagnosis based on WHO criteria; LTBI was defined as immunological evidence of infection without clinical or radiological signs of active disease; sensitivity and specificity were extracted as reported in the original studies, recognizing variability in reference standards.

This review is subject to several limitations, including potential publication bias, restriction to English-language studies, and heterogeneity in diagnostic definitions and reference standards across included studies. Additionally, the absence of a formal meta-analysis limits the ability to provide pooled estimates of diagnostic performance.

## 3. Immunological Basis of Diagnostic Tests

Understanding the immunological mechanisms underlying diagnostic tests for TB is essential for interpreting their performance, particularly in pediatric populations where immune responses differ significantly from those of adults [4,6]. All three tests considered in this review—TST, IGRA, and TBST—are based on the host immune response to Mtb, but they differ in antigen composition, mode of antigen delivery, kinetics of response, and readout of immune activation (Table 1).

In children, the immune system is still developing, with age-dependent differences in T-cell function, cytokine production, and antigen presentation. These factors directly influence the sensitivity and reliability of immunodiagnostic tests. Neonates and young children exhibit reduced Th1 responses, lower interferon-gamma (IFN- γ) production, and a higher likelihood of anergy, all of which may impair test performance [18].

### 3.1. Tuberculin Skin Test (TST)

The TST relies on a delayed-type hypersensitivity (DTH) reaction mediated by antigen-specific T lymphocytes [18,19]. Following intradermal injection of purified protein derivative (PPD), antigen-presenting cells such as dendritic cells process and present mycobacterial antigens via major histocompatibility complex (MHC) class II molecules to CD4+ T cells. In sensitized individuals, memory Th1 cells are activated and release pro-inflammatory cytokines, including IFN-γ, tumor necrosis factor-alpha (TNF-α), and interleukin-2 (IL-2). These cytokines promote recruitment of macrophages and other immune cells, leading to localized inflammation and induration. The magnitude of this reaction reflects prior immune sensitization but does not distinguish between latent infection, active disease, or past exposure.

Several immunological limitations affect TST performance [18,19]. First, the antigenic composition of PPD is highly heterogeneous, containing shared epitopes across multiple mycobacterial species. This leads to cross-reactivity in individuals vaccinated with BCG or exposed to environmental non-tuberculous mycobacteria (NTM), thereby reducing specificity. Second, repeated TST administration may induce a boosting phenomenon, where prior exposure enhances subsequent responses independent of new infection.

In pediatric populations, TST sensitivity is influenced by immune maturity, nutritional status, and co-infections. Infants and young children often exhibit weaker DTH responses, increasing the risk of false-negative results. Additionally, severe or disseminated TB may paradoxically suppress immune responses, further complicating interpretation [18,19].

### 3.2. Interferon-Gamma Release Assays (IGRAs)

IGRAs are in vitro assays designed to measure antigen-specific T-cell responses by quantifying interferon-γ production after stimulation with Mtb-specific antigens [6,10,18,19]. These antigens, primarily ESAT-6 and CFP-10, are encoded in the RD1 region of the Mtb genome and are absent in BCG strains and most NTM, conferring high specificity.

In QuantiFERON assays, whole blood is incubated with antigens, and IFN-γ concentration is measured using ELISA [18]. In T-SPOT.TB, peripheral blood mononuclear cells are isolated and the number of IFN-γ-secreting T cells is quantified using ELISPOT technology [18]. These methods assess circulating effector T-cell responses, providing a snapshot of systemic immune sensitization.

From an immunological standpoint, IGRA responses depend on both the frequency and functional capacity of antigen-specific T cells. In young children, these parameters may be reduced due to immune immaturity, leading to lower sensitivity and higher rates of indeterminate results [18,19]. Factors such as lymphopenia, HIV infection, and acute illness can further impair assay performance [20,21,22,23,24,25].

Another important consideration is that IGRAs primarily detect circulating immune responses, which may not fully reflect localized immune activity at sites of infection such as lymph nodes or lung tissue [20,21,22,23,24,25]. Additionally, pre-analytical variables—including time to incubation, temperature control, and sample handling—can significantly influence results [20,21,22,23,24,25].

### 3.3. Tuberculosis Skin Test (TBST)

The TBST represents a hybrid diagnostic approach that integrates the immunological advantages of antigen specificity with the physiological context of in vivo immune activation [13,19]. TBST employs recombinant ESAT-6 and CFP-10 proteins administered intradermally, triggering a DTH response similar to TST but with improved antigenic precision.

Following injection, antigen-presenting cells process the recombinant proteins and present them to memory T cells, initiating a localized Th1-mediated immune response [13,14]. This results in cytokine release, cellular infiltration, and measurable induration at the injection site.

One key immunological advantage of TBST is the amplification of immune responses in vivo. Even when circulating T-cell responses are weak—as may occur in young children—the localized environment of the skin may facilitate sufficient immune activation to generate a detectable reaction [26,27,28,29]. This may partly explain the improved performance of TBST compared to IGRA in some pediatric studies [26,27,28,29].

Furthermore, the use of RD1 antigens minimizes cross-reactivity with BCG and most NTM, significantly improving specificity compared to TST [30]. Unlike IGRA, TBST is less affected by pre-analytical variables and does not require laboratory processing, making it more robust in real-world settings [13,14].

However, TBST still depends on intact cell-mediated immunity. Conditions such as severe malnutrition, HIV infection, or very early age may reduce responsiveness [26,27,28,29,30]. Additionally, like TST, TBST requires a return visit for reading and is subject to inter-observer variability [13,14].

### 3.4. Immunological Dynamics in Pediatric TB

Pediatric TB is characterized by distinct immunopathological features compared to adult disease. Children are more likely to develop primary progressive disease and disseminated forms such as miliary TB or TB meningitis [31,32,33,34]. These forms are often associated with altered immune responses, including impaired granuloma formation and dysregulated cytokine profiles.

The balance between protective immunity and disease progression is influenced by age, genetic factors, nutritional status, and co-infections [31,32,33,34]. These variables affect the performance of all immunodiagnostic tests and must be considered when interpreting results.

Importantly, none of the current immunological tests—including TBST, TST, and IGRA—can reliably distinguish between LTBI and active disease, as they all measure immune sensitization rather than bacterial burden or replication.

### 3.5. Comparative Immunological Framework

A comparative analysis [30] highlights key differences:

- Antigen composition: TST uses complex, non-specific antigen mixtures; TBST and IGRA use defined RD1 antigens.
- Immune compartment assessed: TST and TBST evaluate in vivo tissue-level responses; IGRA assesses circulating T cells.
- Sensitivity to immune immaturity: IGRA may be more affected by reduced circulating T-cell function, while TBST may partially overcome this through in vivo amplification.
- Specificity determinants: Cross-reactivity affects TST but is minimized in TBST and IGRA.

These immunological distinctions provide a mechanistic explanation for observed differences in diagnostic performance and support the rationale for TBST as a potentially optimal compromise between TST and IGRA in pediatric populations.

## 4. Diagnostic Performance in Pediatric Populations

Evaluating the diagnostic performance of immunological tests in pediatric TB is inherently complex due to the absence of a definitive gold standard, the paucibacillary nature of disease, and the variability of immune responses across age groups [4,7]. In children, microbiological confirmation is frequently not achievable, and composite clinical reference standards are often used, introducing heterogeneity in reported estimates of sensitivity and specificity [30]. Despite these challenges, comparative studies provide important insights into the relative performance of the TST, IGRAs, and the TBST (Table 2).

### 4.1. Sensitivity

Sensitivity is a critical parameter in pediatric TB diagnosis, as delayed or missed diagnoses can result in rapid disease progression and severe complications, particularly in children under five years of age. However, estimating sensitivity remains difficult because microbiological confirmation is often lacking in pediatric cohorts.

Available evidence suggests that TBST demonstrates sensitivity comparable to IGRA [13,14,15] and, in some settings, superior to TST. In children with microbiologically confirmed TB, TBST sensitivity has been reported to range between approximately 75% and 90%, depending on study design and epidemiological context [26,27,28,29,30]. IGRA sensitivity in similar populations generally ranges from 70% to 85%, while TST sensitivity tends to be lower and more variable, especially in younger children and immunocompromised patients [18,19,20,21,22].

Age-dependent immune immaturity plays a major role in test performance. In infants and young children, reduced Th1 responses and lower IFN-γ production can impair all immunological tests [18]. Notably, TBST may retain relatively better sensitivity in this group due to its in vivo mechanism, which allows localized amplification of immune responses at the skin level [26,27,28,29]. In contrast, IGRA performance may be more affected by reduced circulating T-cell responses, resulting in higher rates of indeterminate or false-negative results [18].

In severe or disseminated TB, such as miliary TB or TB meningitis, sensitivity of all immunological tests may be reduced due to immune dysregulation or anergy [18,30,34]. This highlights the importance of integrating test results with clinical and radiological findings.

### 4.2. Specificity

Specificity is essential to avoid overdiagnosis and unnecessary treatment, particularly in screening programs and low-incidence settings [8,9]. TST specificity is significantly affected by prior BCG vaccination and exposure to environmental mycobacteria, leading to false-positive results [35,36].

Both IGRA and TBST use Mtb-specific antigens (ESAT-6 and CFP-10), resulting in substantially improved specificity. TBST specificity has been reported to exceed 90–95% in BCG-vaccinated populations, closely approximating that of IGRA [26,27,28,29,30]. This represents a major advantage over TST, particularly in countries with universal BCG vaccination policies.

However, specificity estimates may be influenced by the definition of uninfected populations. In high-burden settings, distinguishing true negatives from individuals with subclinical or recent infection remains challenging [30].

### 4.3. Concordance and Discordance Between Tests

Studies comparing TBST, TST, and IGRA have reported variable levels of concordance [18,19,20,21,22,23,24,25,26,27,28,29,30]. Agreement between TBST and IGRA is generally higher than between TST and IGRA, reflecting their shared antigenic specificity [30].

Discordant results are frequently observed, particularly in BCG-vaccinated children. TST-positive/IGRA-negative patterns are common and likely reflect cross-reactivity [18,19,20,21,22]. Conversely, TBST-positive/IGRA-negative results may indicate differences between in vivo and ex vivo immune responses [26,27,28,29].

In pediatric populations, discordance may also be influenced by developmental immunology. For example, a child may generate a localized skin response (TBST positive) while lacking sufficient circulating T-cell activity to produce a positive IGRA result [26,27,28,29]. The clinical significance of these discordant patterns remains an important area for further investigation.

### 4.4. Performance in BCG-Vaccinated Populations

BCG vaccination significantly affects TST interpretation, particularly in recently vaccinated children, leading to reduced specificity and diagnostic uncertainty [18,19].

TBST overcomes this limitation by employing RD1-specific antigens absent from BCG strains [13,14]. Multiple studies have demonstrated that TBST maintains high specificity in BCG-vaccinated populations, making it particularly useful in regions with routine BCG immunization [26,27,28,29,30].

IGRA shares this advantage but is often less accessible due to higher costs and infrastructure requirements [18,19,20,21,22].

### 4.5. Performance in Immunocompromised Children

Immunocompromised children—including those with HIV infection, malnutrition, or chronic illnesses—represent a high-risk group in whom diagnostic accuracy is often reduced. All immunological tests rely on functional cell-mediated immunity, which may be impaired in these populations [18,19,20,21,22,23,24,25,26,27,28,29,30].

Both TST and IGRA have demonstrated reduced sensitivity in immunocompromised children. IGRA may also produce higher rates of indeterminate results due to insufficient immune response [18,19,20,21,22,23,24,25]. Evidence regarding TBST performance in immunocompromised children is emerging and remains limited; current data are insufficient to draw firm conclusions [26,27,28,29,30].

### 4.6. Predictive Value and Clinical Utility

The clinical utility of these tests depends not only on sensitivity and specificity but also on predictive values, which are influenced by disease prevalence [37,38]. In high TB-burden settings, positive predictive value increases; however, distinguishing latent infection from active disease remains a fundamental limitation.

Importantly, none of the currently available immunological tests—including TBST—can reliably differentiate between latent infection and active TB [38]. These tests should therefore be interpreted as indicators of immune sensitization rather than definitive diagnostic tools.

### 4.7. Overall Assessment

Overall, TBST appears to provide a favorable balance between sensitivity and specificity in pediatric populations [26,27,28,29,30]. Its performance is comparable to IGRA while maintaining the operational simplicity of TST. These characteristics make TBST a promising diagnostic tool, particularly in BCG-vaccinated and resource-limited settings.

Nevertheless, variability across studies, limited data in key subpopulations, and the absence of standardized evaluation frameworks highlight the need for further large-scale, prospective research.

## 5. Operational Considerations

Operational characteristics are critical determinants of the real-world utility of diagnostic tests, particularly in pediatric TB, where testing often occurs in resource-limited, high-burden settings [11,38]. Beyond analytical performance, factors such as feasibility, cost, infrastructure requirements, training needs, and patient acceptability strongly influence the implementation and scalability of TST, IGRA, and TBST [39].

### 5.1. Ease of Use and Feasibility

TBST shares the same administration technique as TST, involving intradermal injection and measurement of induration after 48–72 h [26,27,28,29,30]. This procedural similarity represents a major advantage, as healthcare workers in many settings are already trained in TST administration and interpretation. As a result, TBST can be integrated into existing workflows with minimal additional training.

In contrast, IGRAs require venipuncture, careful sample handling, and laboratory-based processing within strict time windows [18,19,20,21,22,23,24,25]. These requirements limit feasibility in primary care settings, rural areas, and regions with limited laboratory infrastructure. In pediatric populations, blood collection may also be more technically challenging and less acceptable to both children and caregivers.

However, both TST and TBST require a second patient visit for result reading, which can reduce completion rates and lead to loss to follow-up, particularly in mobile or underserved populations [18,19,20,21,22,23,24,25,26,27,28,29].

### 5.2. Cost and Cost-Effectiveness

Cost considerations are central to diagnostic strategy decisions, especially in low- and middle-income countries with a high TB burden. TST remains the least expensive test, but its lower specificity can lead to overdiagnosis and unnecessary treatment, increasing overall healthcare costs [35].

IGRAs are substantially more expensive due to laboratory requirements, proprietary reagents, and equipment costs. These factors limit their widespread adoption in resource-constrained settings [18].

TBST occupies an intermediate position, offering improved specificity over TST while remaining significantly less expensive and less resource-intensive than IGRA [30]. Preliminary economic analyses suggest that TBST may be cost-effective in BCG-vaccinated populations by reducing false-positive diagnoses and unnecessary preventive therapy [30]. However, comprehensive cost-effectiveness studies across diverse settings are still needed.

### 5.3. Infrastructure and Logistics

Infrastructure requirements differ markedly between tests. TST and TBST require minimal equipment and can be performed in primary healthcare settings without laboratory support [38]. This makes them particularly suitable for decentralized TB programs.

IGRAs, by contrast, require laboratory facilities, temperature-controlled environments, and timely sample processing (typically within 8–16 h) [18]. Delays or improper handling can compromise test validity. These logistical constraints pose significant barriers in many high-burden regions.

Supply chain considerations also play a role. Availability of TBST reagents may currently be limited in some countries, potentially restricting access despite its theoretical advantages [30].

### 5.4. Training and Standardization

Accurate administration and interpretation of skin tests require trained personnel [39]. Variability in intradermal injection technique and measurement of induration can affect both TST and TBST results. Standardized training programs and quality assurance measures are therefore essential.

IGRA results are less operator-dependent at the clinical level but require trained laboratory personnel and standardized procedures to ensure reliability [18].

Inter-observer variability remains a challenge for skin-based tests [39]. Digital measurement tools or standardized reading protocols may help reduce variability in future implementations.

### 5.5. Patient Acceptability and Practical Considerations

Patient acceptability is particularly important in pediatric populations. Skin tests (TST and TBST) are generally well tolerated, though they require two visits and may cause mild discomfort at the injection site [18,19,20,21,22,23,24,25,26,27,28,29,30].

IGRAs require blood sampling, which may be distressing for children and logistically more difficult, particularly in younger age groups [18,19,20,21,22,23,24,25]. However, they have the advantage of requiring only a single visit.

Caregiver preferences, access to healthcare facilities, and local healthcare practices all influence the acceptability and completion rates of different testing strategies.

### 5.6. Programmatic Implementation and Scalability

From a public health perspective, scalability is a key consideration. TST has long been the backbone of TB screening programs due to its simplicity and low cost, but its limitations reduce effectiveness in BCG-vaccinated populations [39].

TBST has the potential to replace or complement TST in many settings, offering improved specificity without substantial increases in operational complexity [26,27,28,29]. Its integration into national TB programs would require regulatory approval, procurement systems, and updated clinical guidelines.

IGRAs are more suitable for high-resource settings or targeted use (e.g., screening in low-incidence countries), but their scalability in high-burden regions remains limited [18,19,20,21,22,23,24,25].

### 5.7. Summary of Operational Considerations

Overall, TBST combines many of the operational advantages of TST with improved diagnostic specificity, making it a promising tool for widespread use in pediatric TB screening and diagnosis. While logistical challenges such as the need for a return visit remain, TBST offers a favorable balance between feasibility, cost, and performance.

Future implementation efforts should focus on ensuring availability, standardizing training, and evaluating real-world cost-effectiveness across diverse healthcare settings.

## 6. Comparison of TBST, TST, and IGRA

A comparison of TBST, TST, and IGRA highlights key differences in antigen specificity, mode of immune response assessment, diagnostic performance, and operational characteristics in pediatric populations [6,8]. TST uses purified protein derivative, a heterogeneous antigen mixture that limits specificity, particularly in BCG-vaccinated individuals. In contrast, TBST and IGRA rely on M. tuberculosis–specific antigens (ESAT-6 and CFP-10), underpinning their higher specificity [18,30].

TBST and IGRA differ in how immune responses are measured: IGRA assesses ex vivo interferon-γ production by circulating T cells, whereas TBST evaluates an in vivo delayed-type hypersensitivity response. This distinction may be relevant in children, where localized in vivo responses could partially compensate for reduced circulating T-cell activity, although evidence remains variable [18,19,20,21,22,23,24,25,26,27,28,29,30].

In terms of diagnostic performance, TBST shows specificity comparable to IGRA and higher than TST, with sensitivity generally similar to IGRA and variable across studies. None of the tests can distinguish latent infection from active disease [18,19,20,21,22,23,24,25,26,27,28,29,30].

Operationally, TST and TBST are low-cost, skin-based tests requiring minimal infrastructure but a second visit, whereas IGRA requires laboratory capacity and blood sampling but only one visit. TBST may represent a pragmatic balance between improved specificity and field applicability, particularly in BCG-vaccinated and resource-limited settings [18,35,39].

Overall, TBST can be considered a hybrid approach combining key advantages of TST and IGRA. While promising, its role in pediatric TB diagnosis requires further validation in diverse settings. Table 3 shows the advantages and limitations of the three tests.

## 7. Clinical and Public Health Implications

The introduction of TBST into clinical practice and public health strategies has the potential to significantly influence the diagnosis, management, and control of pediatric TB [1,39]. Given the persistent challenges associated with diagnosing TB in children, particularly in high-burden settings, improved immunodiagnostic tools are essential to reduce morbidity, mortality, and transmission [36].

From a clinical perspective, TBST may enhance diagnostic confidence in situations where TST results are difficult to interpret, especially in BCG-vaccinated children [26,27,28,29,30]. The higher specificity of TBST reduces the likelihood of false-positive results, thereby minimizing unnecessary treatment with potentially toxic anti-tubercular drugs [30]. This is particularly relevant in pediatric populations, where overtreatment carries important risks, including hepatotoxicity, poor adherence, and psychological burden for both patients and caregivers.

At the same time, maintaining adequate sensitivity is crucial to avoid missed diagnoses. TBST appears to offer a balance between sensitivity and specificity that may improve clinical decision-making, particularly when used as part of a comprehensive diagnostic approach that includes clinical assessment, radiological findings, and epidemiological context [30]. In this regard, TBST should not be viewed as a standalone diagnostic tool, but rather as a component of integrated diagnostic algorithms.

In the context of LTBI, TBST may play an important role in identifying children at risk of disease progression who would benefit from preventive therapy [30]. Improved specificity compared to TST is particularly advantageous in this setting, as it reduces unnecessary treatment in children who are not truly infected. This has implications not only for individual patient care but also for healthcare resource utilization.

From a public health perspective, the implementation of TBST could strengthen TB control programs, particularly in countries with widespread BCG vaccination [39]. By reducing false-positive diagnoses associated with TST, TBST may improve the efficiency of screening programs and allow for more targeted use of preventive therapy. This is especially important in high-burden settings, where resources are limited and prioritization is essential.

TBST may also facilitate decentralization of TB diagnostic services [39]. Its similarity to TST in terms of administration allows it to be deployed in primary healthcare settings without the need for laboratory infrastructure, unlike IGRA. This feature enhances accessibility and supports early diagnosis, which is critical for interrupting transmission chains and preventing disease progression in children.

In low-incidence, high-resource settings, TBST could serve as an alternative to IGRA in certain scenarios, particularly when laboratory access is limited or rapid screening is required [39]. However, the relative advantages of TBST versus IGRA in these settings will depend on cost-effectiveness analyses, local epidemiology, and healthcare system organization.

Implementation of TBST at scale will require careful consideration of regulatory approval, procurement systems, training of healthcare personnel, and integration into national and international guidelines. Standardization of test administration and interpretation will be essential to ensure consistent performance across different settings.

Despite its promise, several challenges remain. Evidence on TBST performance in key populations, such as immunocompromised children and those under two years of age, is still limited [26,27,28,29,30]. In addition, long-term outcomes associated with TBST-guided clinical decisions, such as progression from LTBI to active disease, require further investigation.

Ultimately, the adoption of TBST has the potential to bridge the gap between the simplicity of TST and the specificity of IGRA, offering a pragmatic and scalable solution for improving pediatric TB diagnosis [26,27,28,29]. Its successful integration into clinical practice and public health strategies will depend on continued research, policy support, and alignment with global TB control priorities.

## 8. Future Directions

The continued development and evaluation of the TBST represent an important priority in advancing the diagnosis of pediatric TB [38]. While current evidence suggests that TBST offers a favorable balance between specificity, sensitivity, and operational feasibility, several key knowledge gaps must be addressed to fully establish its role within clinical practice and global TB control strategies.

A major area for future research is the need for large, well-designed, multicenter prospective studies conducted across diverse epidemiological settings. Such studies should include high-burden and low-incidence regions, as well as populations with varying levels of BCG coverage and exposure to non-tuberculous mycobacteria. Standardized study designs and harmonized reference definitions would help reduce heterogeneity and allow for more robust comparisons between TBST, TST, and IGRA.

Particular attention should be given to the performance of TBST in vulnerable pediatric subgroups. These include very young children, especially those under two years of age, in whom immune responses are still developing, as well as immunocompromised populations such as children living with HIV, those with severe malnutrition, or those receiving immunosuppressive therapies. Understanding TBST performance in these groups is essential, as they are at highest risk of disease progression and adverse outcomes.

Another critical research priority is the evaluation of TBST within longitudinal cohort studies. Such studies would allow assessment of the prognostic value of TBST, including its ability to predict progression from latent tuberculosis infection to active disease. This information is particularly important for guiding preventive therapy strategies and optimizing risk stratification in pediatric populations.

Comparative effectiveness research is also needed to determine how TBST performs within real-world diagnostic algorithms. Future studies should evaluate combinations of TBST with clinical, radiological, and microbiological tools to identify optimal diagnostic pathways. Additionally, head-to-head comparisons with IGRA in different clinical scenarios would help clarify whether TBST can replace or complement existing tests.

Economic evaluations will play a central role in informing policy decisions. Comprehensive cost-effectiveness analyses should consider not only the direct costs of testing but also downstream consequences, including unnecessary treatment, healthcare utilization, and long-term outcomes. These analyses should be tailored to different epidemiological and healthcare system contexts.

Implementation science will be crucial for translating research findings into practice. Studies should explore barriers and facilitators to TBST adoption, including regulatory approval processes, supply chain considerations, training requirements, and acceptability among healthcare providers and patients. Integration of TBST into national TB programs and international guidelines will require coordinated efforts and evidence-based policy development.

Technological innovation may further enhance the utility of TBST. Advances in antigen formulation, delivery systems, and digital tools for measuring and interpreting skin test reactions could improve accuracy and reduce inter-observer variability. Additionally, research into novel biomarkers and host-response signatures may complement TBST and contribute to more precise diagnostic approaches.

Finally, future work should align with global TB elimination goals, including the WHO End TB Strategy [39]. The development and deployment of improved diagnostic tools such as TBST must be integrated within broader efforts to strengthen health systems, expand access to care, and reduce disparities in TB diagnosis and treatment among children.

In summary, while TBST represents a promising advancement in pediatric TB diagnostics, its full potential will only be realized through continued research, rigorous evaluation, and thoughtful integration into clinical and public health frameworks.

## 9. Conclusions

Pediatric TB remains a major global health challenge, with diagnostic limitations related to the low specificity of TST in BCG-vaccinated populations and the operational constraints of IGRAs. These gaps highlight the need for accurate and feasible diagnostic tools.

TBST has emerged as a promising alternative, combining the antigenic specificity of IGRA with the simplicity of TST. Current evidence suggests high specificity comparable to IGRA and sensitivity similar to, or in some settings higher than, TST, particularly in BCG-vaccinated children. Its low infrastructure requirements and compatibility with existing workflows support its potential for use in resource-limited and decentralized settings.

However, TBST shares key limitations with existing tests, including the inability to distinguish latent from active disease and reduced performance in some high-risk groups. Evidence remains heterogeneous, and data in important subpopulations are still limited.

Overall, TBST represents a promising but not yet definitive tool for pediatric TB diagnosis. It is best considered within integrated diagnostic approaches, with further research needed to clarify its role in clinical practice and public health strategies.

## Figures and Tables

**Table 1:** Immunological characteristics.

Feature	TST	IGRA	TBST
Antigens	PPD (mixed)	ESAT-6, CFP-10	ESAT-6, CFP-10
Specificity	Low (BCG affected)	High	High
Immune response	In vivo DTH	Ex vivo IFN-γ	In vivo DTH
Sample type	Skin	Blood	Skin
Affected by BCG	Yes	No	No

BCG, Bacillus Calmette–Guérin; DTH, Delayed-Type Hypersensitivity; IGRA, interferon-gamma release assay; TBST, tuberculosis skin test; TST, Tuberculin Skin Test.

**Table 2:** Diagnostic performance.

Parameter	TST	IGRA	TBST
Sensitivity	65–80%	70–85%	75–90%
Specificity	80–94%	88–96%	90–95%
<5 years	Reduced	Reduced	Better
Immunocompromised	Reduced	Reduced	Uncertain

IGRA, interferon-gamma release assay; TBST, tuberculosis skin test; TST, Tuberculin Skin Test.

**Table 3:** Advantages and limitations.

Test	Advantages	Limitations
TST	Low cost, widely available	Low specificity
IGRA	High specificity	Expensive, lab-dependent
TBST	High specificity, field-friendly	Two visits

IGRA, interferon-gamma release assay; TBST, tuberculosis skin test; TST, Tuberculin Skin Test.

## Data Availability

No new data were created or analyzed in this study. Data sharing is not applicable to this article.
